# Simultaneous Integrated Boost Volumetric Modulated Arc Therapy for Rectal Cancer: Long-Term Results after Protocol-Based Treatment

**DOI:** 10.1155/2022/6986267

**Published:** 2022-04-07

**Authors:** Dong Soo Lee, Seong-Taek Oh, Chang-Hyeok Ahn, Jaeim Lee, Kil-Yong Lee, Hiun Suk Chae, Sung Soo Kim, Sang Woo Kim, Kyung Jin Seo

**Affiliations:** ^1^Department of Radiation Oncology, College of Medicine, The Catholic University of Korea, Seoul, Republic of Korea; ^2^Department of Surgery, College of Medicine, The Catholic University of Korea, Seoul, Republic of Korea; ^3^Department of Internal Medicine, College of Medicine, The Catholic University of Korea, Seoul, Republic of Korea; ^4^Department of Hospital Pathology, College of Medicine, The Catholic University of Korea, Seoul, Republic of Korea

## Abstract

**Background:**

Volumetric modulated arc therapy (VMAT) with simultaneous integrated boost (SIB) is an advanced form of radiotherapy (RT) technology. The purpose of this study was to report long-term treatment outcomes in patients with locally advanced rectal cancer undergoing VMAT-SIB based concurrent chemoradiotherapy (CRT).

**Methods:**

Between January 2016 and January 2018, a total of 22 patients with operable stage II-III rectal adenocarcinoma were recruited for the pre-designed VMAT-SIB RT protocol. All patients underwent standard diagnostic and staging work-up. The RT target volumes included the following areas: PTV1 = mesorectum that contained gross tumors and enlarged lymph node regions and PTV2 = mesorectum and regional lymphatics from L4-5/S1 to 3-4 cm below the tumor or levator ani muscle, excluding PTV1. The VMAT-SIB dose prescription was as follows: PTV1 = 52.5 Gy/daily 2.1 Gy/25 fractions, PTV2 = 45 Gy/daily 1.8 Gy/25 fractions.

**Results:**

The mean age of the study population was 64 (range, 18-84) years, and 15 (68.2%) patients were male. Radical operation (total mesorectal excision) was performed by either low anterior resection, ultralow anterior resection, or abdominal perineal resection. All five (22.7%) of the patients with confirmed increasing serum carcinoembryonic antigen (CEA) level at diagnosis showed normalization of serum CEA level after the planned treatment. Among 20 patients who underwent preoperative CRT and surgery, tumor down staging in T- and N-stages was achieved in 10 patients (50%) and 13 patients (65%), respectively, with 20% of ypT0/Tis. With a median follow-up of 54.2 (range, 22.6-61.1) months, the 5-year disease-free survival, overall survival, and local control rates were 64.6%, 81.8%, and 84.4%, respectively. Five patients developed distant metastasis and one developed local recurrence as a first event. Two cases with anastomosis site leakage, three with adhesive ileus, and two with abscess formation were observed during postoperative periods.

**Conclusions:**

The current VMAT-SIB-based CRT protocol provided acceptable treatment and toxicity outcomes.

## 1. Introduction

Rectal cancer persists as one of the most frequently diagnosed cancers and a leading cause of cancer-related death worldwide [1, 2]. Treatment paradigms have evolved with the advent of state-of-the-art radiation therapy (RT) technologies, as well as more sophisticated surgical techniques and more efficacious chemotherapeutic agents [3–7]. Neoadjuvant chemoradiation therapy (CRT) followed by total mesorectal excision (TME) surgery and postoperative adjuvant chemotherapy is the current standard of care for patients with locally advanced rectal cancer [8–10].

With respect to the technical aspects of RT, several controversial issues have been discussed. Although some studies have failed to show any significant benefit in terms of reduced toxicity or improved oncologic outcomes of intensity-modulated radiation therapy (IMRT), more recent studies have demonstrated dosimetric superiority of IMRT, which can be translated into better local control and lower toxicity [11–15].

Volumetric modulated arc therapy (VMAT), a novel IMRT approach, allows continuous RT delivery as the gantry rotates around the patient. During the operation, dose rate (fluence), speed of gantry rotation, and shape of multi-leaf collimator can be continuously adjusted and changed to attain highly conformal IMRT delivery [16–18].

In addition, the simultaneous integrated boost (SIB) technique, which can administer individualized RT dose prescriptions concomitantly, can be accompanied with VMAT and can improve the advantage of IMRT [7, 19].

In this study, we constructed a VMAT-SIB protocol as a neoadjuvant RT regimen that can provide slightly higher doses to the high-risk regions [gross tumor and lymph node (LN) areas] and slightly lower doses to the moderate-risk regions [mesorectum and regional lymphatics]. The purpose of this study was to elucidate treatment outcomes and follow-up results after applying the protocol-based treatment.

## 2. Materials and Methods

### 2.1. Study Population and Patient Eligibility

Between January 2016 and January 2018, a total of 22 patients with operable stage II-III rectal adenocarcinoma were recruited to the prospectively designed VMAT-SIB RT protocol. All patients underwent standard diagnostic and staging work-up, including sigmoidoscopy or colonoscopy, abdomino-pelvic magnetic resonance imaging (MRI), abdomino-pelvic and chest computed tomography (CT) scans, positron emission tomography computed tomography (PET-CT) scan, and serum laboratory measurements (complete blood count, hepatic and renal function tests, tumor marker tests).

All patients were histologically confirmed with newly diagnosed locally advanced rectal adenocarcinoma (cT3-T4 and/or cN+) and tumors were located within 12 cm from the anal verge. All patients were ≥18 years of age; had adequate performance status (Eastern Cooperative Oncology Group performance score 0-2) with no evidence of distant metastasis; and had adequate bone marrow, renal, and hepatic function.

Although protocol-based VMAT-SIB preoperative CRT followed by standard surgery was proposed for the entire study population, patients who refused further surgery after initial CRT were also included in this analysis.

This study was approved by the Catholic Medical Center Ethics Committee (approval No: UC21RISI0137) and was in agreement with the Declaration of Helsinki.

### 2.2. Radiation Therapy Protocol

The patients were referred to the department of radiation oncology after completing the entire diagnostic and staging work-up. For the simulation, all patients were immobilized in the supine position with a comfortably full bladder and underwent contrast-enhanced planning CT with 3-mm-thick slices from at least the L3/L4 junction to 2 cm below the perineum using a CT simulator (SOMATOM Definition AS+, Siemens Medical Solutions USA, Inc.). The image datasets were transferred to the Eclipse treatment planning system (ECLIPSE™, Varian Medical Systems). Re-simulation was routinely performed in the entire study population for cone down (CD) plans after 20 fractions.

Target volumes were defined according to the International Commission on Radiation Units & Measurements (ICRU) reports 50, 62, and 83 [20–22]. The gross tumor volume (GTV) was defined as all gross disease (tumor and LN) visible on CT and MRI. The clinical target volume (CTV)1_tumor was generated by expanding 0.3 cm circumferentially and 1.5 cm craniocaudally from the GTV, ensuring that the entire mesorectum and presacral region were encompassed at involved levels. The CTV1_node was generated by expanding 0.5 cm circumferentially and 1 cm craniocaudally from the GTV. The CTV2 includes the entire mesorectum and regional lymphatics (internal iliac, obturator, presacral, and mesorectal) from L4-5/S1 to 2-3 cm below the tumor or levator ani muscle excluding the CTV1 areas. External iliac nodes were included when substantial involvement of genitourinary structures was noted, and the anal canal and ischiorectal fossa were included when levator ani muscle or anal canal involvement was suspected. Planning target volume (PTV)1 was the CTV1 plus 0.2-0.3 cm circumferentially and 0.5-1 cm craniocaudally. PTV2 was the CTV2 plus 0.2-0.3 cm circumferentially and 0.5-1 cm craniocaudally. In summary, the PTV1 covered the mesorectum that contained gross tumors and enlarged lymph node (LN) regions, and the PTV2 covered the entire mesorectum and regional lymphatic areas from L4-5/S1 to 3-4 cm below the tumor or levator ani muscle, excluding PTV1. The GTV and CTV1 were modified in the CD plans according to the re-simulation CT, and the craniocaudal length of mesorectal regions in CTV2 was reduced around any tumors with 1.5-2 cm margins. All organs at risk (OARs) were contoured including the bladder, female and male urogenital organs, both femoral heads, and small/large intestinal loops.

The planned VMAT-SIB dose prescription was as follows: PTV1 = 52.5 Gy/daily 2.1 Gy/25 fractions, PTV2 = 45 Gy/daily 1.8 Gy/25 fractions.

**Figure 1:**
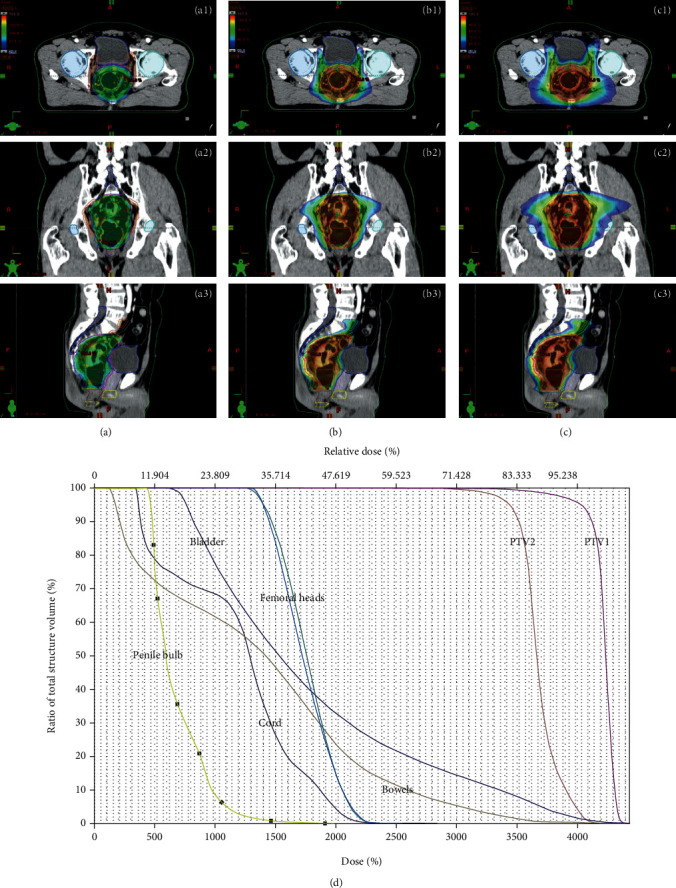
A representative case of RT plan and dose volume histogram. Axial (1), coronal (2), and sagittal (3) view in A (95% isodose lines of PTV1), B (70% isodose lines of PTV1), and C (50% isodose lines of PTV1) are shown. D indicates dose volume histograms of targets and organs at risk.

All patients underwent planned therapy with the VMAT technique using two arcs of RapidArc in the Eclipse treatment planning system (ECLIPSE™, Varian Medical Systems) and plans were optimized for PTV and OAR dose constraints used in the RTOG trial [12]. For treatment verification, megavoltage cone beam CT scans were acquired daily and fused with the planning CT scans prior to treatment. A representative case of the RT plan and dose volume histogram is illustrated in Figure 1.

### 2.3. Chemotherapy

Chemotherapy was administered with either capecitabine or 5-FU/leucovorin (LV). For cases that received capecitabine, concurrent chemotherapy was planned to begin on the first day of RT and continue until the end of the RT course. Capecitabine (1650 mg/m^2^) was prescribed orally twice daily five days a week from Monday to Friday. For cases that received 5-FU/LV, two cycles of intravenous bolus 5-FU (400 mg/m2) and LV (20 mg/m2) were planned for five days during the first and fifth weeks of RT during CRT and an additional four cycles after surgery.

Adjuvant chemotherapy was delivered after surgery in 10 patients who received capecitabine, and the most commonly used regimen was 4-6 cycles of 5-FU/LV.

### 2.4. Surgery

Surgery with TME was scheduled at 8-12 weeks after completion of CRT. The types of surgery and performance of a temporary ileostomy were determined by the surgeons. The pathological stage was recorded based on the American Joint Committee on Cancer 7th edition guidelines [23] and the tumor response was graded according to the tumor regression grade based on the criteria of Dworak et al. [24, 25].

### 2.5. Toxicity Evaluation and Follow-up

Toxicities and adverse events were assessed and recorded every week during CRT according to the Common Terminology Criteria for Adverse Events (CTCAE) version 4.0. Patient follow-up was conducted one month after completion of CRT and every 1-3 months thereafter. Patient history collection and physical examination were routinely conducted at each follow-up, along with a toxicity evaluation. Laboratory and imaging studies were performed when clinically indicated.

### 2.6. Study Endpoints and Statistics

The study endpoints were disease-free survival (DFS), local control (LC), and overall survival (OS) rates. DFS was estimated from the date of diagnosis to the date of disease recurrence, death from any cause, or the last follow-up. LC rate was defined as the proportion of control in the primary site. OS was estimated from the date of diagnosis to the date of death from any cause or to the last follow-up.

Survival curves were generated using the Kaplan-Meier methods. Statistical analyses were performed using the statistical software R, version 4.0.2 (R Foundation for Statistical Computing, Vienna, Austria) and SPSS statistics version 18.0 (SPSS, Inc., Chicago, IL, USA).

## 3. Results

### 3.1. Patient and Tumor Characteristics

Baseline patient and tumor characteristics are described in Table 1. The majority of the study population was male (*n* =15, 68.2%) and had an Eastern Cooperative Oncology Group (ECOG) performance status scale score of 0 (*n* =16, 72.7%). An abnormal serum carcinoembryonic antigen (CEA) level (>7) was found in five (22.7%) patients, two in cT3N0, one in cT3N1, and two in cT3N2. A total of 12 patients had history of medical comorbidities including cardiovascular, pulmonary, endocrine, and neoplastic diseases. The neoplastic diseases included urothelial carcinoma in situ in one case, thyroid papillary cancer in one, and both endometrial and ovarian cancer in one. No recurrence or cancer-related death was observed during follow-up.

### 3.2. Treatment Characteristics

The treatment characteristics for the 20 patients who underwent preoperative CRT and curative surgery are summarized in Table 2. The total RT dose was 52.5 Gy in 25 fractions in all 20 patients. The majority of the study population received xeloda chemotherapy (*n* =18, 90%). The mean preoperative CRT duration and preoperative CRT-surgery interval were 35.3 days and 2.3 months, respectively. Most of the study population underwent laparoscopic low anterior resection (*n* =12, 60%) followed by ultra-low anterior resection (*n* =4, 20%). Pathological findings indicated tumor down-staging in T- and N-stages in 10 (50%) and 13 patients (65%), respectively, with 20% of ypT0/Tis (Table 3). Serum CEA level was normalized in the entire study population. In pathological review, LN extracapsular extension (ECE) was noted in all four ypN(+) patients. Detailed pathological tumor characteristics after chemoradiation are described in Table S1.

Two elderly patients refused additional curative surgery after completing CRT; one was an 82-year-old female patient who received 56.7 Gy of total RT, and the other was a 79-year-old male patient who received 52.5 Gy of total RT.

### 3.3. Treatment Outcomes

A total of six (27.3%) recurrences and four (18.2%) deaths occurred during the median follow-up of 54.2 (range, 22.6-61.1) months. The two-year and five-year DFS rates were 81.8% and 64.6%, respectively. Among six recurrences, five cases and one case developed distant metastasis (DM) and local recurrence (LR) as a first event, respectively. The most common distant relapse site was the lung (4) followed by the brain (1). The patient, tumor, and treatment characteristics of recurrent cases are depicted in Table 4.

The two-year and five-year LC rates were 100% and 84.4%, respectively. All LRs (*n* =2) were categorized as clinically suspicious, and there were no histologically proven cases. Of these cases, the initial types of surgery were abdominoperineal resection (APR) and total proctocolectomy. One LR developed at 21 months after an initial event of DM as a second relapse. Two clinically suspicious LR cases are shown in Figure S1.

**Figure 2:**
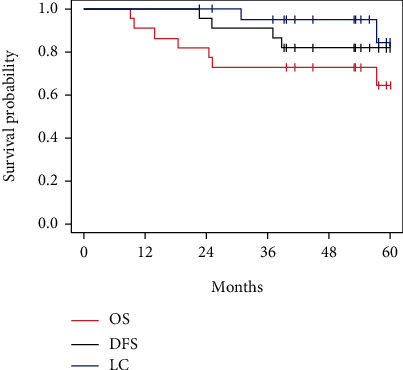
The Kaplan-Meier curves of LC, DFS, and OS.

The two-year and five-year OS rates were 95.5% and 81.8%, respectively. Among six patients who relapsed, four died with causes attributable to disease, and two survived after successful salvage treatments. The two patients who survived were managed by salvage targeted therapy/chemotherapy and stereotactic ablative lung radiotherapy. The Kaplan-Meier curves of LC, DFS, and OS are shown in Figure 2.

Of the two patients who refused curative surgery, one died 25.1 months later due to an unknown cause at a convalescence hospital, and the other was followed for 54.2 months without recurrence. Based on the final imaging studies, the tumors were in a regressed state in both patients.

### 3.4. Treatment-Related Toxicities

During the CRT course, the toxicities were mild (G0-2) and the patients recovered with conservative care. During the postoperative periods, two anastomosis site leakages, three adhesive ileuses, and two abscess formations were observed. Anastomosis site leakages developed at 1.7 and 18.3 months after surgery. Of the two cases, one resolved after several years and the other went through APR due to fistula development. All adhesive ileus cases presented in the early postoperative period (0.5, 2.6, and 0.4 months, respectively) and resolved thereafter except for patient who experienced repeated ileus. In that patient, anastomotic stricture developed 11.2 months after surgery and was treated with T-loop colostomy. Abscesses were resolved after proper conservative care, including antibiotic treatment.

## 4. Discussion

This study showed that tumor responses and LC rates, as well as toxicity profiles, were comparable to those in historical series [7, 10, 11, 26–29]. More recently, contemporary trials have incorporated advanced RT techniques and more intensified systemic agents and presented promising results [4, 7, 29–31]. RT dose escalation using innovative technologies has enabled improved tumor responses with equivalent toxicity outcomes, albeit insufficient long-term results [4, 7, 29, 32]. Alongi et al. described pathological complete response (pCR) rate of 17.5% without grade 3 or higher toxicities among patients treated with IMRT boosting to 60 Gy in 30 fractions [32]. Cubillo et al. examined patients who were treated with 57.5 Gy by SIB-IMRT and reported a 50% pCR rate [4]. In comparison, even after higher doses were delivered to the tumor by concomitant boost IMRT, and high rates of pCR were achieved, a high incidence of LR rates was reported. A promising pCR rate of 23.7% with a treatment regimen of 55 Gy via IMRT was reported by Zhu et al. [7]. However, the three-year local recurrence rate was 14.6% and grade 3 radiation dermatitis rate was 17.9% in a median 30 months of follow-up. In the current study, LR cases developed even after aggressive surgeries (one APR and one total proctocolectomy) in relatively late periods (30.8 and 57.3 months). Moreover, all cases were not biopsy-proven, clinically suspicious lesions. Therefore, the role of CRT as a neoadjuvant regimen was not inadequate in our protocol.

A number of prognostic factors have been addressed in rectal cancer patients who underwent preoperative CRT and surgery [33–35]. Our study did not focus on identification of established prognostic factors. Although adverse effects of LN ECE status in a variety of solid tumors have been reported [36–38], their role in rectal cancer remains unclear [39–41]. In the present study, all four ypN(+) patients showed LN ECE in pathological review. Among them, two died of disease after DM development, and the remaining two were alive without evidence of recurrence. Although additional study results are required, our results are in agreement with Lino-Silva et al. [40] and the emerging role of LN ECE in rectal cancer needs to be established.

The incidence of main failures has changed due to the introduction of advanced surgical (TME) and RT technologies [42, 43]. Our study also showed that DM (83.3%) was the main pattern of failure and surpassed the incidence of LR. Therefore, integration of more potent systemic agents and further identification of novel biomarkers are strongly recommended [7, 30, 44, 45].

VMAT technique has technical advantages in terms of greater conformal and homogeneous dose distribution and shorter beam delivery time as compared to multi-field step-and-shoot IMRT techniques [13, 16–18]. Previous dosimetric comparative studies have shown that prone positioning can significantly reduce OAR dose, particularly in small bowel even in an IMRT setting [15, 46]. However, we treated patients in the supine position to maintain the daily set-up consistency. Nonetheless, toxicity profiles demonstrated that our set-up technique did not compromise outcomes. Recently, topical endorectal administration and intraperitoneal spacer injection of hyaluronic acid have been attempted to prevent radiation proctitis and will be implemented if beneficial effect is demonstrated in the VMAT era [47, 48].

The watch-and-wait strategy is emerging as a new treatment option for patients that achieve clinical CR after CRT [8, 49]. Our study also showed that two elderly patients achieved a disease-free status at 25.1- and 54.2-month follow-up even though they did not undergo surgery after CRT. Therefore, selection of the most suitable candidates for this organ preservation approach needs to be explored in future clinical trials.

The distinctive strengths of this study were demonstration of long-term results after the VMAT-SIB protocol and the feasibility of target volume conformation by creating relatively narrow margins around the mesorectum. The pre-designed VMAT-SIB protocol was uniformly adapted in the entire study population. Very high doses were not prescribed to the high-risk regions due to concerns for toxicities. Because TME surgery was planned, relatively narrow circumferential margins around the mesorectum were generated to reduce unnecessary perioperative toxicities. Of note, the LC outcomes for our protocol were acceptable, with two-year and five-year LC rates of 100% and 84.4%, respectively. However, the main patterns of failure (DM) and several late toxicities imply further room for improvement in future treatments. The relatively small sample sizes and utilization of heterogeneous chemotherapeutic agents are additional limitations of this study.

In summary, our VMAT-SIB-based CRT protocol was well tolerated and yielded favorable long-term oncologic results in patients with locally advanced rectal cancer. Development of more optimized dose-prescription schedules as neoadjuvant RT regimens and combining novel systemic agents might be crucial to elicit improved outcomes in future cohorts.

## Figures and Tables

**Table 1:** Baseline patient and tumor characteristics.

Characteristics		*N* (%)
Age		
Mean ± SD	64 ± 15.4	
Gender		
	Male	15 (68.2)
	Female	7 (31.8)
ECOG PS		
	0	16 (72.7)
	1	5 (22.7)
	2	1 (4.5)
cT-stage		
	cT2	1 (4.5)
	cT3	17 (77.3)
	cT4a	1 (4.5)
	cT4b	3 (13.6)
cN-stage		
	cN0	4 (18.2)
	cN1	11 (50)
	cN2	7 (31.8)
CEA level		
Mean ± SD	15.7 ± 53.4	
	≤7	17 (77.3)
	>7	5 (22.7)
Tumor location (anal verge, cm)		
Mean ± SD	4.8 ± 2.4	
	≤3	6 (27.3)
	>3	16 (72.7)

ECOG PS: Eastern Cooperative Oncology Group performance status; CEA: carcinoembryonic antigen.

**Table 2:** Treatment characteristics.

Characteristics		*N* (%)
Total preop RT dose		
	52.5 Gy	20 (100)
Concurrent chemotherapy		
	Xeloda	18 (90)
	5-fluorouracil/leucovorin	2 (10)
Preop CRT duration (days)		
Mean ± SD	35.3 ± 1.7	
Preop CRT-OP interval (months)		
Mean ± SD	2.3 ± 0.5	
OP title		
	Lap LAR	12 (60)
	Lap uLAR	4 (20)
	APR	3 (15)
	Total proctocolectomy	1 (5)

RT: radiation therapy; CRT: chemoradiation; OP: operation; LAR: low anterior resection; uLAR: ultra-low anterior resection; APR: abdominoperineal resection.

**Table 3:** Pathological analysis after chemoradiation.

Characteristics		*N* (%)
ypT-stage		
	ypT0/tis	3/1 (20)
	ypT1	2 (10)
	ypT2	3 (15)
	ypT3	10 (50)
	ypT4b	1 (5)
ypN-stage		
	ypN0	16 (80)
	ypN1a	1 (5)
	ypN1b	2 (10)
	ypN2	1 (5)
Resection margin		
	Wide	20 (100)
	Close (<1 mm) or positive	0 (0)
Tumor regression grade		
	1	3 (15)
	2	8 (40)
	3	6 (30)
	4	3 (15)
Lymphatic invasion		
	Negative	19 (95)
	Positive	1 (5)
Venous invasion		
	Negative	19 (95)
	Positive	1 (1)
Perineural invasion		
	Negative	17 (85)
	Positive	3 (15)

**Table 4:** Detailed characteristics of recurrent cases.

Patients	Age	Sex	cTN	ypTN	CEA	Location (AV)	Surgery type	DFI (m)	Recur site(s)	Final status
1	76	M	T3N1	T3N1a	3	10	LAR	18.5	Multiple brain	DWD
2	64	M	T4bN2	T3N0	2.7	1	APR	9.8	Multiple lung->SB, presacral	DWD
3	84	M	T3N2	T3N0	9.8	4	Total proctocolectomy	57.3	Presacral	DWD
4	68	M	T3N2	T3N0	1.9	6	LAR	9.1	Mediastinal/hilar LNs	NED
5	77	M	T3N0	T2N0	1.3	3	LAR	24.5	Lung (oligorecurrence)	NED
6	68	F	T3N1	T3N2	1	7	LAR	13.8	Multiple lung, paraaortic LNs	DWD

CEA: carcinoembryonic antigen; AV: anal verge; DFI: disease-free interval; M: male; LAR: low anterior resection; APR: abdominoperineal resection; DWD: die with disease; SB: small bowel; LN: lymph node; NED: no evidence of disease; F: female.

## Data Availability

All data are available from the corresponding author upon request.

## Supplementary Materials

Supplementary 1 Figure S1. Two clinically suspicious local recurrence cases in our study. A-1/B-1 (initial) and A-2/B-2 (recurrence) are shown.

Supplementary 2 Table S1. Pathological tumor characteristics after chemoradiation
