# Supplementary figures and images for: Simultaneous Integrated Boost Volumetric Modulated Arc Therapy for Rectal Cancer: Long-Term Results after Protocol-Based Treatment
Source: J Oncol. 2022 Apr 7;2022:6986267. doi: 10.1155/2022/6986267 (PMC9012974; doi:10.1155/2022/6986267)

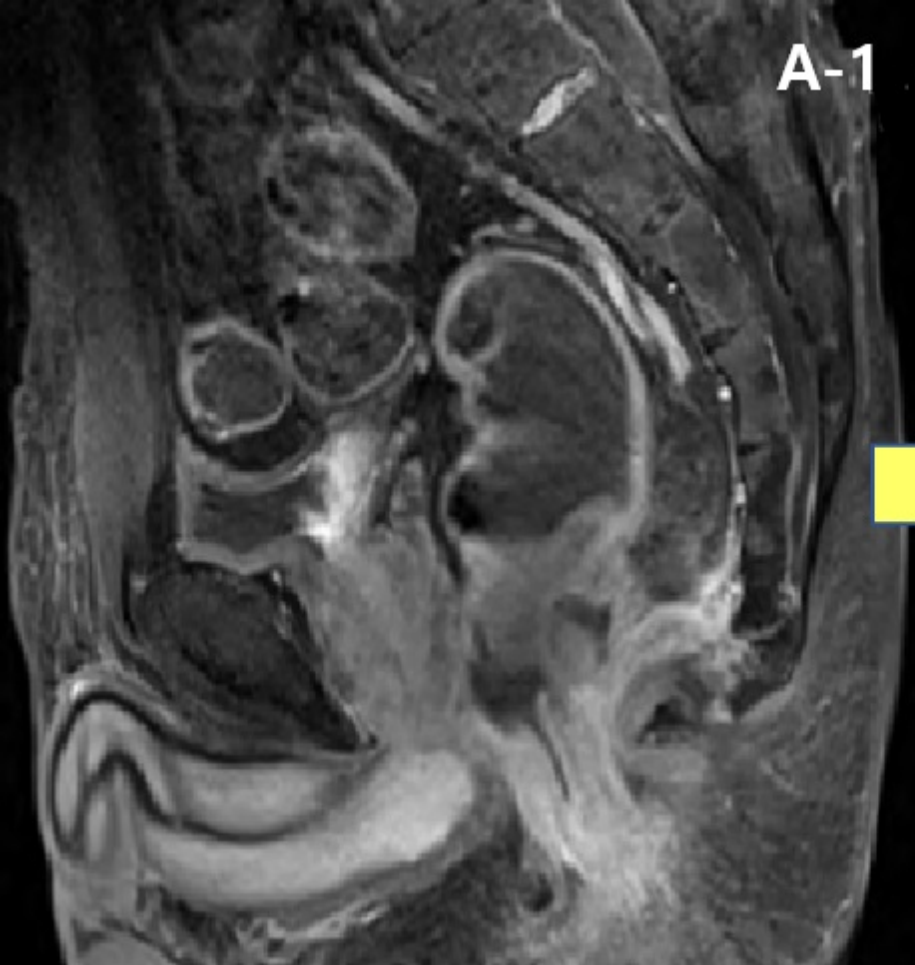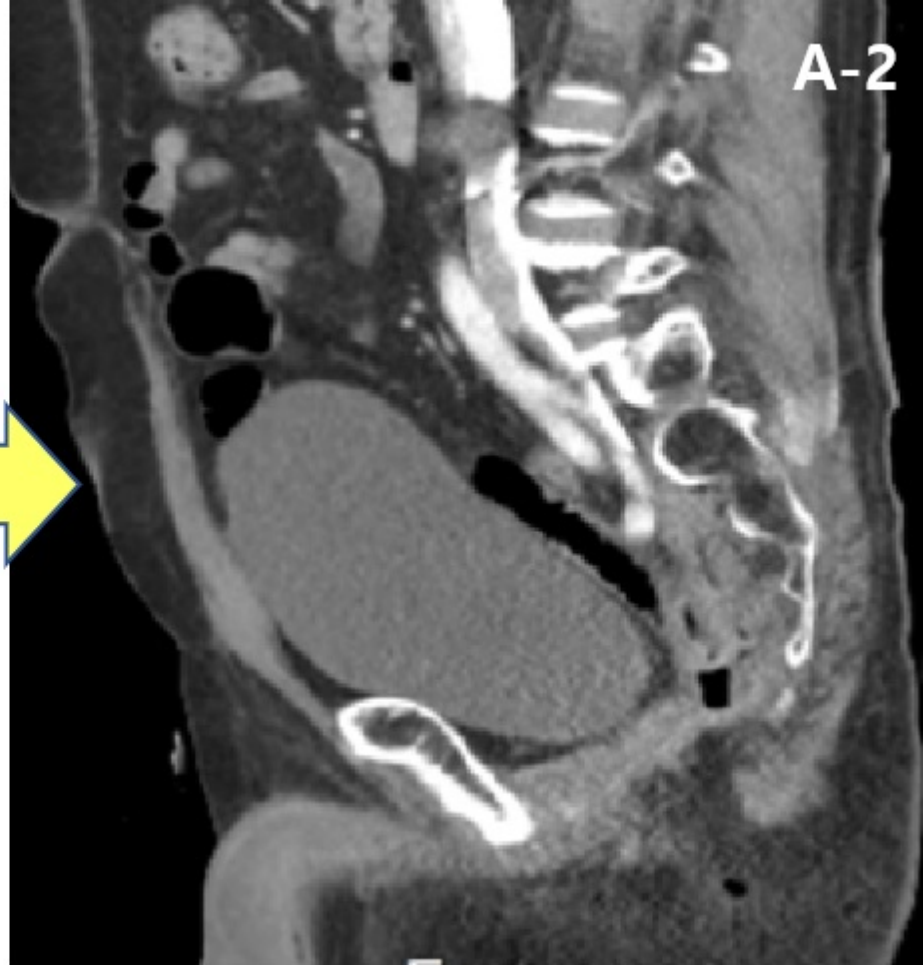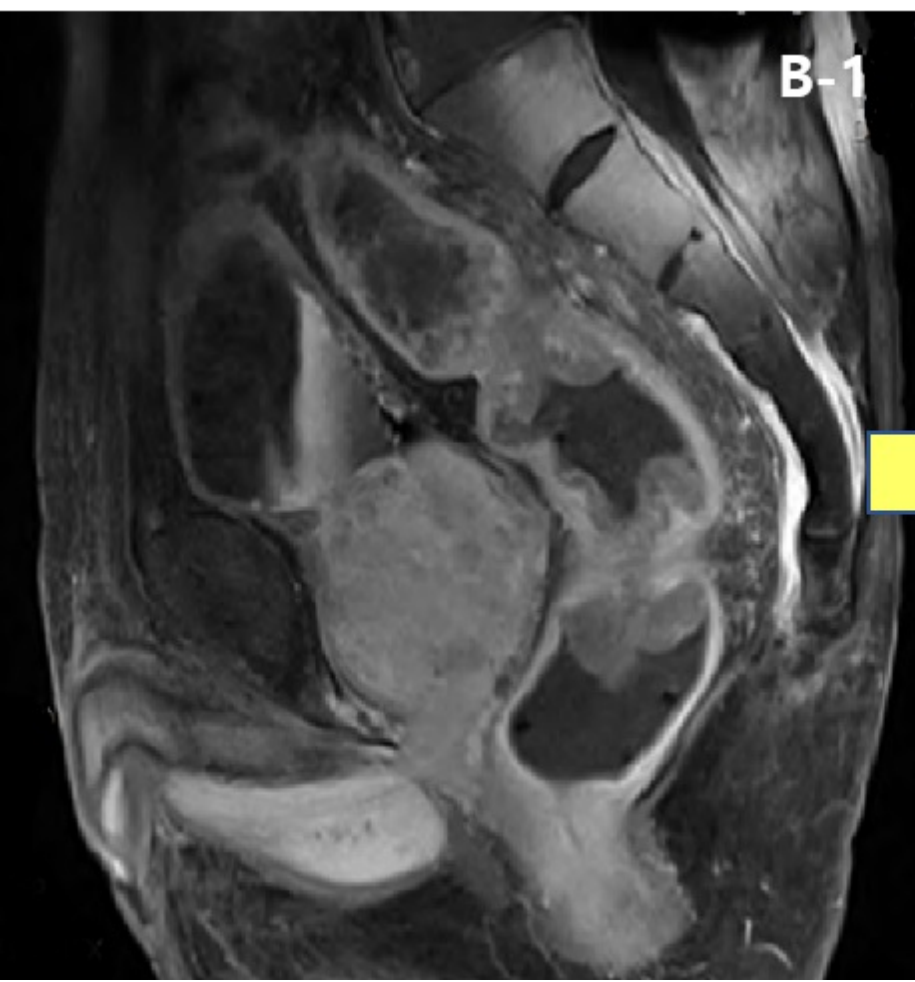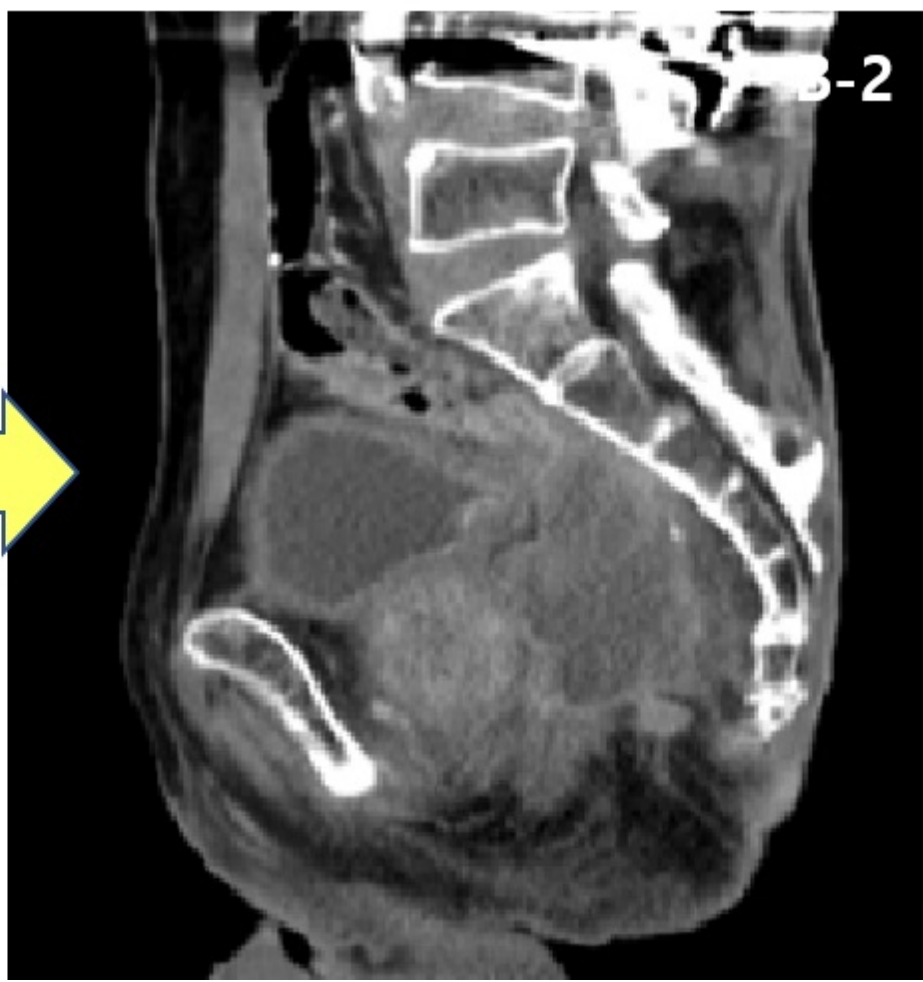

Supplement: Supplementary 1 — Figure S1. Two clinically suspicious local recurrence cases in our study. A-1/B-1 (initial) and A-2/B-2 (recurrence) are shown. [file 6986267.f1.pdf]
