# Supplementary material for: Simultaneous Integrated Boost Volumetric Modulated Arc Therapy for Rectal Cancer: Long-Term Results after Protocol-Based Treatment
Source: J Oncol. 2022 Apr 7;2022:6986267. doi: 10.1155/2022/6986267 (PMC9012974; doi:10.1155/2022/6986267)
Supplement: Supplementary 2 — Table S1. Pathological tumor characteristics after chemoradiation [file 6986267.f2.docx]

Table S1. Pathological tumor characteristics after chemoradiation

| Patients | cTN | ypTN | Tumor grade | TRG | LN ECE | LI | VI | PNI | MSI | K-RAS mut | Recur |
| --- | --- | --- | --- | --- | --- | --- | --- | --- | --- | --- | --- |
| 1 | T3N1 | T3N0 | MD | 3 |  | - | - | - | - | - |  |
| 2 | T3N1 | T4bN1b | WD | 1 | + | + | + | + | - | - |  |
| 3 | T4bN2 | T3N1b | PD | 2 | + | - | - | - | - | - |  |
| 4 | T4aN2 | TisN0 | WD | 3 |  | - | - | - | - | + |  |
| 5 | T3N1 | T3N1 | MD | 2 | + | - | - | ++ | - | - | Distant |
| 6 | T2N1 | T2N0 | WD | 1 |  | - | - | - | - | - |  |
| 7 | T3N0 | T0N0 | MD | 4 |  | - | - | - | - | - |  |
| 8 | T4bN2 | T3N0 | MD | 2 |  | - | - | + | - | + | Distant->Local |
| 9 | T3N1 | T3N0 | MD | 2 |  | - | - | - | - | + |  |
| 10 | T3N2 | T3N0 | MD | 2 |  | - | - | - | - | - | Local |
| 11 | T3N2 | T3N0 | MD | 3 |  | - | - | - | - | - | Distant |
| 12 | T3N1 | T1N0 | MD | 3 |  | - | - | - | unknown | unknown |  |
| 13 | T3N0 | T2N0 | MD | 3 |  | - | - | - | unknown | unknown |  |
| 14 | T3N2 | T0N0 | MD | 4 |  | - | - | - | - | + |  |
| 15 | T3N1 | T3N0 | MD | 2 |  | - | - | - | - | + |  |
| 16 | T3N0 | T2N0 | WD | 2 |  | - | - | - | - | - |  |
| 17 | T3N1 | T3N0 | MD | 2 |  | - | - | - | - | unknown | Distant |
| 18 | T3N1 | T3N2 | MD | 1 | + | - | - | - | + | + |  |
| 19 | T3N2 | T0N0 | WD | 4 |  | - | - | - | - | unknown | Distant |
| 20 | T3N0 | T1N0 | WD | 3 |  | - | - | - | - | + |  |

TRG, Tumor regression grade; LN ECE, Lymph node extracapsular extension; LI, Lymphatic invasion; VI, Vascular invasion; PNI, Perineural invasion; MSI, Microsatellite instability; MD, Moderate differentiated; WD, Well differentiated; PD, Poorly differentiated.
